# Exploring the potentials of sorghum genotypes: a comprehensive study on nutritional qualities, functional metabolites, and antioxidant capacities

**DOI:** 10.3389/fnut.2023.1238729

**Published:** 2023-08-10

**Authors:** Sukyeung Lee, Yu-Mi Choi, Myoung-Jae Shin, Hyemyeong Yoon, Xiaohan Wang, Yoonjung Lee, Jungyoon Yi, Young-ah Jeon, Kebede Taye Desta

**Affiliations:** ^1^International Technology Cooperation Center, Technology Cooperation Bureau, Rural Development Administration, Jeonju, Republic of Korea; ^2^National Agrobiodiversity Center, National Institute of Agricultural Sciences, Rural Development Administration, Jeonju, Republic of Korea

**Keywords:** antioxidant activity, anthocyanidins, nutrition, fatty acids, flavonoids, protein, *Sorghum bicolor*

## Abstract

**Introduction:**

Sorghum, long regarded as one of the most underutilized crops, has received attention in recent years. As a result, conducting multidisciplinary studies on the potential and health benefits of sorghum resources is vital if they are to be fully exploited. In this study, the nutritional contents, functional metabolites, and antioxidant capacities of 23 sorghum breeding lines and three popular cultivars were assessed.

**Materials and method:**

All of the sorghum genotypes were grown under the same conditions, and mature seeds were hand-harvested. The metabolite contents and antioxidant capacities of sorghum seeds were assessed using standard protocols. Fatty acids were quantified using a gas chromatography-flame ionization detector, whereas flavonoids and 3-deoxyanthocyanidins were analyzed using a liquid chromatography-tandem mass spectrometry method. The data were analyzed using both univariate and multivariate statistical approaches.

**Results and discussion:**

Total protein (9.05–14.61%), total fat (2.99–6.91%), crude fiber (0.71–2.62%), dietary fiber (6.72–16.27%), total phenolic (0.92–10.38 mg GAE/g), and total tannin (0.68–434.22 mg CE/g) contents varied significantly across the sorghum genotypes (*p* < 0.05). Antioxidant capacity, measured using three assays, also differed significantly. Five fatty acids, including palmitic, stearic, oleic, linoleic, and linolenic acids, were found in all the sorghum genotypes with statistically different contents (*p* < 0.05). Furthermore, the majority of the sorghum genotypes contained four 3-deoxyanthocyanidins, including luteolinidin, apigeninidin, 5-methoxyluteolinidin, and 7-methoxyapigeninidin, as well as two dominant flavonoids, luteolin and apigenin. Compared to the cultivars, some breeding lines had significantly high levels of metabolites and antioxidant activities. On the other hand, statistical analysis showed that total tannin, total phenolic, and antioxidant capacities varied significantly across white, yellow, and orange genotypes. Principal component analysis was used to differentiate the sorghum genotypes based on seed color and antioxidant index levels. Pearson’s correlation analysis revealed strong links between biosynthetically related metabolites and those with synergistic antioxidant properties.

**Conclusion:**

This research demonstrated the diversity of the sorghum resources investigated. Those genotypes with high levels of nutritional components, functional metabolites, and antioxidant activities could be used for consumption and breeding programs.

## 1. Introduction

Sorghum (*Sorghum bicolor* (L.) Moench) is one of the most widely grown cereal crops in the world. Sorghum, known for its drought tolerance, stress resistance, low production cost, and high yield, can be grown in water-stressed, arid, and semi-arid environments throughout Africa, Asia, America, and Europe (1–4). The latest Food and Agriculture Organization of the United Nations (FAO) data shows that global sorghum production reached approximately 61 million tonnes in 2021, covering approximately 41 million ha of cultivable land (5). In many Asian, South American, and African countries, the crop is a primary source of nutrition and energy, and it is consumed in a variety of forms such as bread, porridge, and popped snacks (1). It was primarily grown for animal feed in Western countries, but due to its high natural ingredient contents, there is a growing interest in using it for human consumption and biofuel production (3).

Previous studies have found that sorghum seeds are high in both nutritional and non-nutritional metabolites. Vitamins, protein and amino acids, oil and fatty acids, minerals, and fiber are among the nutritional metabolites reported, and they are important in promoting the use of sorghum seeds in different food industries (6, 7). Sorghum can also be used as a biofuel feedstock, which is essential for renewable energy production (8). Furthermore, several targeted and untargeted metabolite profiling studies identified different classes of polyphenols in sorghum seeds, including anthocyanins (in particular 3-deoxyanthocyanidins), phenolic acids, and flavonoids (9–11). *In vitro* and *in vivo* studies have also revealed that these metabolites contribute to the various pharmacological properties of sorghum seeds, including antioxidant, anticancer, anti-inflammatory, and anti-diabetic properties (3, 12–14). Additionally, Rezaee et al. (15) conducted a recent review that focused on the potential of sorghum polyphenols in preventing Alzheimer’s disease. Sorghum seeds are also gluten-free, making them an excellent choice for people suffering from celiac disease or gluten intolerance (16, 17).

Although sorghum is still one of the underutilized crops, new studies have expanded to include sorghum-based food items aimed for human consumption (7). Darman et al. (18), for example, examined the nutritional composition of sorghum-based biscuits and reported considerable levels of carbohydrates, dietary fiber, and energy. Another study reported improved chemical, functional, and nutritional properties of sorghum-based snacks fortified with mung bean (19). Furthermore, Abdelhalim et al. (20) reported high levels of total phenolic content (88.7 mg GAE/g) and total tannin content (9.2 mg CE/g), as well as strong antioxidant properties, in a sorghum-only beverage. Several recent reviews provide detailed information on a wide variety of sorghum-based functional foods (7, 21). In general, these previous studies further signify the importance of sorghum resources in the production of more sustainable dietary products. In another aspect, recent advances in genomics and breeding technologies have reignited interest in sorghum breeding to speed up the development of improved varieties (2, 22). As a result, selecting and utilizing sorghum resources such as landraces, cultivars, and breeding lines with optimal nutritional quality, functional components, and bioactivities, among others, is crucial (23, 24). Furthermore, genetic and environmental factors affect the metabolite compositions and contents of sorghum seeds. Therefore, the performance of sorghum resources should be assessed regularly in order to utilize those with desirable characteristics (2, 25).

Several studies assessed the nutritional contents and metabolite levels of various sorghum resources as described before (4, 26–28). Many of these studies focused on a specific class of metabolite in a single and/or small number of sorghum resources at a time. Investigating diverse types of sorghum metabolites utilizing a variety of resources leads to a greater understanding of their metabolite compositions, diversity, and potential as future foods. In this study, we cultivated 23 sorghum breeding lines alongside 3 cultivars. Then, the levels of nutritional components (total protein, total fat, crude fiber, dietary fiber, and five dominant fatty acids), functional metabolites (total tannin content, total phenolic content, three individual flavonoids, and four individual 3-deoxyanthocyanidins), and antioxidant capacity (using three independent assays) were assessed. Furthermore, the relative performance of the breeding lines in comparison to the cultivars was assessed. The findings could pave the way for other studies that support the use of sorghum for consumption as well as in developing improved varieties.

## 2. Materials and methods

### 2.1. Chemicals and reagents

The chemicals and reagents used in this study were of analytical grade (purity >99.8%) and were used as obtained. HPLC-grade water, acetonitrile, methanol, and sulfuric acid were purchased from Fisher Scientific (Pittsburgh, PA, United States). The remaining chemicals and reagents, including luteolin, apigenin, luteolinidin, apigeninidin, eriodictyol, catechin, gallic acid, L-ascorbic acid, formic acid, anhydrous sodium carbonate (Na_2_CO_3_), vanillin, Folin–Ciocalteu phenol reagent, potassium ferricyanide, trichloroacetic acid, ferric chloride, 1,1-diphenyl-2-picrylhydrazyl (DPPH) radical, 2,2′-azino-bis(3-ethylbenzothiazoline-6-sulfonic acid) diammonium salt (ABTS), and 6-hydroxy-2,5,7,8-tetramethylchroman-2-carboxylic acid were ordered from Sigma-Aldrich (St. Louis, MO, United States).

### 2.2. Seed materials and sample preparation

The seeds of the 23 sorghum breeding lines and 3 commonly grown cultivars were received from the gene bank at the National Agrobiodiversity Center, Rural Development Administration (Jeonju, Republic of Korea). The seeds were sown on June 17, 2021 in an experimental field found at the center (latitude/longitude: 30°49′38.37″ N/127°09′7.78″ E), and the cultivation period lasted until October of the same year. The mature seeds were hand-harvested, and seed samples from each genotype were dried in a post-harvest crop dryer (TJHP-1003, Jungang Jeongmil, Korea) at 40°C for 7 days. The samples were then ground using a grinder (Geno Grinder, SPEX, Metuchen, NJ, United States), placed in sealed plastic bags and kept at −20°C pending extraction and subsequent analysis. The color of mature sorghum seeds was also determined by visually grading roughly 10 seeds per genotype with a QP card 203 color code (Supplementary Figure S1).

### 2.3. Extract preparation for total tannin content, total phenolic content, and antioxidant activities

Polyphenols were extracted using a previously reported protocol with some modifications (29). Briefly, 1 g of powdered sorghum seed sample was mixed with 5 mL of 80% methanol (aqueous) in a 15 mL electron beam irradiated polypropylene extraction tube (SPL Life Sciences, Gyeonggi-do, Republic of Korea). The mixture was vortexed and extracted in a water bath for 45 min at 25°C using a microprocess-controlled sonicator (PowerSonic 250, Hwashin Tech, Seoul, Republic of Korea). The mixture was then removed and centrifuged for 15 min at 13000 rpm using a high-speed Labogene 1236R centrifuge (Labogene, Daejeon, Republic of Korea), with the supernatant retained. The extraction process was repeated for the residue, and the combined supernatant was used to analyze the total tannin content (TTC), total phenolic content (TPC), and antioxidant activities as briefed below. In each case, analysis was conducted within 72 h after extraction, and samples were stored at -20°C when not used. Moreover, absorbance was determined using an Eon Microplate Spectrophotometer (Bio-Tek, Winooski, VT, United States) during each experiment.

#### 2.3.1. Determination of TTC

The TTC was determined using a method proposed by Price et al. (30) with slight modifications. Initially, a vanillin-HCl reagent was prepared by mixing equal parts methanol solutions of 8% HCl and 1% vanillin. Then, 100 μL of sample extract and 200 μL of the reagent were mixed followed by incubation for 20 min at 25°C in the dark. The absorbance was then determined at 500 nm. Catechin was used as a standard to plot calibration curve (y = 0.0353x + 0.0135, *R*^2^ = 0.9983) at five concentration levels (0.25–8.00 mg/mL). TTC was calculated as milligrams of catechin equivalents per gram on a dry seed weight basis (mg CE/g) using catechin as a standard.

#### 2.3.2. Determination of TPC

The Folin–Ciocalteu method, as modified in our recently reported protocol (31), was used to determine the total phenolic content. Initially, 100 μL of seed extract was mixed with an equal volume of 2 N Folin–Ciocalteu reagent in the dark. Then, the mixture was treated with 100 μL of 2% Na_2_CO_3_ solution after 3 min of incubation at 25°C. Finally, the absorbance at 750 nm was measured after 30 min of reaction in the dark using methanol as a blank. Calibration curve (y = 8.6431x – 0.2476, *R*^2^ = 0.9999) was plotted using gallic acid at five concentration levels (0.025–3.00 mg/mL). TPC was determined as milligrams of gallic acid equivalent per gram on a dry seed weight basis (mg GAE/g).

#### 2.3.3. Determination of 1,1-diphenyl-2-picrylhydrazyl radical (DPPH^•^) scavenging activity

For DPPH^•^ scavenging activity, 100 μL of the sorghum seed extract was mixed with an equivalent volume of freshly made DPPH solution (150 μM), and the mixture was incubated for 30 min in the dark at 25°C. Then, the absorbance at 517 nm was measured, and ascorbic acid was used as a standard. Ascorbic acid was used as a standard to plot calibration curve (y = 4049.3x - 7.7906, *R^2^* = 0.9998) at five concentration levels (0.0025–0.0250 mg/mL). The DPPH^•^ scavenging activity was reported as milligrams of ascorbic acid equivalent antioxidant capacity per gram on a dry seed weight basis (mg AAE/g) (31).

#### 2.3.4. Determination of 2,2′-Azino-bis(3-ethylbenzothiazoline-6-sulfonic acid) diammonium radical cation (ABTS^•+^) scavenging activity

A stock solution of ABTS^•+^ was initially prepared by dissolving ABTS (7 mM) in potassium persulfate (K_2_S_2_O_8_, 2.45 mM). The mixture was incubated for 16 h at 25°C before being diluted with water to achieve an absorbance of 0.700 ± 0.02 at 734 nm. Then, 10 μL of the sorghum seed extract was mixed with 150 μL of ABTS^•+^ solution and the mixture was incubated for 3 min at 25°C in the dark. Finally, the absorbance was measured at 734 nm. Using Trolox as a standard, calibration curve (y = 395.06x – 1.3275, R^2^ = 0.9998) was plotted at five concentration levels (0.005–0.250 mg/mL). The ABTS^•+^ scavenging activity was determined as milligrams of Trolox equivalent antioxidant capacity per gram on a dry seed weight basis (mg TE/g) (31).

#### 2.3.5. Determination of ferric-reducing antioxidant power

Initially, 60 μL of the sorghum seed extract was mixed with 150 μL of freshly prepared phosphate buffer (pH: 6.6, 0.2 M) and 150 μL of 1% potassium ferricyanide solution. The mixture was incubated for 20 min at 50°C, followed by the addition of 150 μL of 10% trichloroacetic acid, and centrifugation at 13,000 rpm for 10 min. Then, 100 μL of the supernatant was mixed with an equal volume of distilled water, followed by the addition of 20 μL of 0.1% ferric chloride solution. The absorbance at 700 nm was measured after 10 min of incubation. Ascorbic acid was used as a standard to plot calibration curve (y = 3.5623x – 0.0033, *R^2^* = 0.9999) at five concentration levels (0.0025–0.0250 mg/mL), and the FRAP activity result was reported in mg AAE/g (31).

### 2.4. Antioxidant index

The antioxidant index (AI) was determined according to the method described by Ng et al. (32) and was used to rank the sorghum genotypes based on their overall antioxidant capacity. In brief, the AI of each sorghum genotype was calculated as the average relative percentage value obtained from the five different colorimetric assays, which included DPPH^•^ scavenging activity, FRAP, ABTS^•+^ activity, TTC, and TPC. In each assay, the highest value was considered 100%, and the remaining lower values were converted using the numerical scale using the equation AI = [(sample score/best score) × 100]. Using the AI values, the sorghum genotypes were classified as very low (0–19%), low (20–39%), medium (40–59%), high (60–79%), or very high (80–100%) AI genetic materials.

### 2.5. Determination of nutritional components

The total protein, total fat, crude fiber, and dietary fiber contents were determined using standard methodologies (33). In summary, the total protein content was calculated as N x 6.25 using the Kjeldahl method, where N is the released nitrogen content and 6.25 is the standard conversion factor. Similarly, the total fat content was determined using the soxhlet extraction method with a Soxtec800 extractor (FOSS, Hillerod, Denmark) and diethyl ether as a solvent, and reported as a percentage of weight loss before and after extraction. The total crude fiber content (in percent) was determined using a Fiber Analyzer (FOSS, Hillerod, Denmark) and the modified Henneberg and Stohmann method. Similarly, the total dietary fiber content was determined using an Analytical Fibertec E-1023 System (FOSS, Hillerod, Denmark) and the enzymatic-gravimetric assay. In each assay, samples were prepared and analyzed in triplicates, and values were reported on a dry seed weight basis.

### 2.6. Identification and quantification of fatty acids

For fatty acid analysis, fatty acid methyl esters (FAMEs) were synthesized using a direct methylation technique as previously described with a minor modification (34). In brief, 0.2 g of powdered sample was added in 10 mL round bottom glass tube with a screw cap and mixed with 680 μL of solvent mixture consisting of methanol, benzene, 2,2-dimethoxypropane, and sulfuric acid in the ratio of 39:20:5:2. Then, 400 μL of n-heptane was added, and the mixture was vortexed for 20 s before extraction in a shaking water bath set at 80°C. After 2 h, the mixture was removed and allowed to cool at 25°C before being centrifuged for 15 min at 13000 rpm. The upper n-heptane layer containing FAMEs was retained, filtered, and put into an injection vial. Then, 1 μL of the sample, at a split ratio of 50:1, was injected into a pre-optimized QP2010 gas chromatography (GC) instrument (Shimadzu, Kyoto, Japan), which was equipped with a flame ionization detector (FID) and an HP-INNOWAX column (30 m × 0.250 mm, 0.25 μm). During separation, the initial column temperature was set at 100°C and gradually increased to 170°C at a rate of 60°C/min. After holding this temperature for 1 min, it was raised to 240°C with a 6.5°C/min ramp and held for another minute. The total analysis took 16.4 min to complete. During analysis, the detector and injection port both had temperatures of 250°C. Helium was used as a carrier gas at a flow rate of 1.5 mL/min. The acquired chromatograms were integrated and analyzed using LabSolution software (Shimadzu, Kyoto, Japan) and the target fatty acids were identified using the retention times of the corresponding standards. The content of each fatty acid was calculated as the relative percent of the total fatty acid using the area of GC-peaks.

### 2.7. Analysis of 3-deoxyanthocyanidins and flavonoids

#### 2.7.1. Extraction of 3-deoxyanthocyanidins and flavonoids

The extraction of 3-deoxyanthocyanidins and flavonoids was performed in methanol containing 1% HCl (29). In a 15 mL polypropylene extraction tube (SPL Life Sciences, Gyeonggi-do, Republic of Korea), 1 g of powdered sorghum seed sample was combined with 5 mL of the solvent and vortexed to homogenize. The mixture was sonicated for 45 min at 25°C in a water bath (PowerSonic 250, Hwashin Tech, Seoul, Republic of Korea). The mixture was then removed, centrifuged at 13000 rpm for 15 min using a high-speed Labogene 1236R centrifuge (Labogene, Daejeon, Republic of Korea), and the supernatant was retained. The extraction process was repeated for the residue. The combined supernatant was filtered through a 0.45 μm micro-membrane filter and made ready for injection.

#### 2.7.2. Identification and quantification of 3-deoxyanthocyanidins and flavonoids

Identification of the target metabolites was achieved using a Waters Acquity ultra-performance liquid chromatography (UPLC) system outfitted with a PDA detector and a Synapt XS Q-TOF mass spectrometer (Waters, Milford, MA, United States). During analysis, 10 μL of sample extract was injected and separation was carried out on a Waters ACQUITY UPLC HSS T3 column (100 × 2.1 mm, 1.8 μm) set to 40°C. As a mobile phase, a binary solvent system of 0.1% formic acid (A) and acetonitrile (B) was used. The solvent flow began with 10% B at a rate of 0.3 mL/min, and the gradient conditions were set as follows: 10–25% B (0–15 min), 25–40% B (15–25 min), and 40–90% B (25–30 min). The final condition was equilibrated to 10% B for the last 5 min (30.1–35 min). The mass scanning range was 100–1,200 Da in both positive and negative ionization modes with capillary voltages of 3 kV and − 2.5 kV, respectively. The collision energy was 20–50 V and the cone voltage was 30 V. The ion source temperature was 120°C, while the desolvation temperature was 450°C. The chromatograms and mass spectra were analyzed using MassLynx 4.1 software, and anthocyanins were read at 500 nm and flavonoids at 360 nm. Molecular mass data, fragment ions (*m/z*), and the UV–Vis spectrum data along with commercially available standards, were used to confirm the identity of the target molecules (Supplementary Scheme S1).

A Nexera UPLC system (Shimadzu, Kyoto, Japan) equipped with a Shimpack GIST C18 column (100 × 2.1 mm, 2.1 μm) under a similar UPLC condition described above was used to quantify the target anthocyanins and flavonoids. Luteolinidin, apigeninidin, luteolin, and apigenin, were quantified using the corresponding external standards. 5-methoxyluteolinidin and 7-methoxyapigeninidin, with no available standards, were quantified as luteolinidin and apigeninidin equivalents, respectively multiplied by the appropriate correction factors (4). In each case, calibration curves were plotted at seven concentration levels of the standards (0.005–1 mg/mL) and the target metabolites were quantified from peak-area responses acquired from the UPLC-chromatograms. The collected data were processed using LabSolution software (Shimadzu, Kyoto, Japan) and the contents of each were reported as milligrams per gram (mg/g) on a dry seed weight basis.

### 2.8. Statistical analysis

For each experiment, measurements were performed in triplicates and results are expressed as mean ± standard deviation (SD). Significant differences between measurements were statistically determined using analysis of variance (ANOVA) at *p* < 0.05 level. The statistical and principal component (PCA) analyzes were carried out using xlstat software (Lumivero, CO, United States). Scatter grams, scatter plots, and Pearson’s correlation analyzes were computed using R-software (version 4.0.2, r-project).

## 3. Results and discussion

Sorghum has received a lot of attention in recent years, and a range of food products derived from its seeds are being developed for dietary purposes (7). As a result, multidisciplinary research emphasizing the potential of sorghum resources in food industries and breeding is crucial. This study looked at the functional metabolites, nutritional characteristics, and antioxidant activities of 23 breeding lines and 3 cultivars. The sorghum genotypes showed significant variations in each of the analyzed metabolites and antioxidant activities. The performance of the breeding lines was also evaluated in relation to the cultivars. The sections that follow provide thorough descriptions of the overall findings.

### 3.1. TTC, TPC, and antioxidant activities

The TTC, TPC, and antioxidant activities of individual sorghum genotypes are provided in Table 1. The TTC and TPC were in the ranges of 0.68–434.22 mg CE/g and 0.29–10.38 mg GAE/g, respectively (*p* < 0.05), with mean values of 186.01 mg CE/g and 4.51 mg GAE/g. Previously, several studies have investigated the total tannin and total phenolic content of several sorghum genetic resources, and the reported values were wide-ranging. The TPC range obtained in our study was consistent with a previous study in which TPC ranged from 1.56 to 11.99 mg GAE/g across 11 sorghum varieties (35). In other research, Rhodes et al. (36) observed a much wider TPC range (0.00 to 37.46 mg GAE/g), whereas Ofosu et al. (11) reported a much smaller TTC range (45.0–98.7 mg CE/100 g). Diverse studies have also documented a wide range of TTC and TPC values in sorghum resources. The observed variations in reported values could be attributed to genetic differences, analysis protocols, and growth conditions (4, 12, 17, 37–39). Many of these studies also investigated the antioxidant activities of sorghum resources although there are discrepancies in analysis protocol and reporting methods. In our study, the DPPH^•^ scavenging activity, ABTS^•+^ scavenging activity, and FRAP all exhibited significant variations, with values ranging from 0.24 to 49.12 mg AAE/g, 1.22 to 49.19 mg TE/g, and 0.40 to 23.10 mg AAE/g, respectively (Table 1). Among the breeding lines, IS8009, which had the highest TTC and the third highest TPC (9.29 mg GAE/g), displayed the highest ABTS^•+^ scavenging activity and FRAP. IS8121, on the other hand, demonstrated the highest DPPH^•^ scavenging activity while having the highest TPC and second highest TTC (414.06 mg CE/g). In contrast, IS12611 displayed the lowest TPC, ABTS^•+^ scavenging activity, and FRAP, while ET 185–2 and ET 36–1 displayed the lowest TTC and DPPH^•^ scavenging activity, respectively. As shown in Table 1, genotypes with high levels of TTC and TPC had pronounced antioxidant activities indicating the synergistic role of such metabolites in regulating reactive oxygen species (ROS) and reactive nitrogen species (RNS) (39, 40).

**Table 1:** Total tannin, total phenolic, antioxidant activities, and their relative (%) levels in sorghum genotypes.

Genotype	Seed color	TTC (mg CE/g, %)	TPC (mg GAE/g, %)	DPPH (mg AAE/g, %)	ABTS (mg TE/g, %)	FRAP (mg AAE/g, %)	AI	
							%	Level
Bonita	White	40.84 ± 0.24^l^, 9.40	2.14 ± 0.03^m^, 20.65	2.52 ± 0.12^n^, 8.57	4.66 ± 0.32^q^, 9.48	1.32 ± 0.05^pq^, 5.73	10.77	VL
Darset	Red	103.83 ± 1.63^j^, 23.91	3.62 ± 0.23^jk^, 34.87	9.97 ± 0.02^k^, 33.84	10.45 ± 0.18^n^, 21.27	5.28 ± 0.08^n^, 22.86	27.35	L
IS8005	Yellow	243.97 ± 26.15^g^, 56.19	8.36 ± 0.45^c^, 80.58	22.15 ± 1.66^d^, 75.16	27.43 ± 1.71^i^, 55.84	15.42 ± 1.02^g^, 66.75	66.90	H
IS8009	Yellow	434.22 ± 22.63^a^, 100.00	9.29 ± 0.23^b^, 89.48	24.11 ± 0.02^c^, 81.81	49.12 ± 0.23^a^, 100.00	23.10 ± 0.52^a^, 100.00	94.26	VH
IS8014	Yellow	378.05 ± 1.25^c^, 87.06	5.42 ± 0.03^gh^, 52.26	24.23 ± 0.18^c^, 82.23	47.56 ± 0.44^bc^, 96.82	21.17 ± 0.43^c^, 91.63	82.00	VH
IS8051	Yellow	307.85 ± 3.03^f^, 70.90	5.25 ± 0.01^h^, 50.59	24.04 ± 0.03^c^, 81.59	43.61 ± 0.10^d^, 88.79	17.69 ± 0.54^f^, 76.58	73.69	H
IS8089	Orange	109.83 ± 0.79^j^, 25.29	2.70 ± 0.01^l^, 26.02	8.87 ± 0.04^l^, 30.08	18.15 ± 0.02^m^, 36.95	5.35 ± 0.18^n^, 23.18	28.31	L
IS8093	Orange	226.50 ± 2.17^h^, 52.16	4.67 ± 0.01^i^, 44.98	20.15 ± 0.38^e^, 68.38	32.38 ± 0.30^h^, 65.91	11.56 ± 0.20^j^, 50.05	56.30	M
IS8107	Yellow	353.30 ± 2.31^d^, 81.36	9.47 ± 0.06^b^, 91.24	28.97 ± 0.04^ab^, 98.31	43.70 ± 0.44^d^, 88.96	18.71 ± 0.14^e^, 80.98	88.17	VH
IS8112	Orange	256.78 ± 1.33^g^, 59.14	5.66 ± 0.04^fg^, 54.56	24.55 ± 0.25^c^, 83.29	35.53 ± 0.31^g^, 72.33	13.83 ± 0.41^h^, 59.88	65.84	H
IS8121	Yellow	414.06 ± 9.77^b^, 95.36	10.38 ± 0.09^a^, 100.00	29.47 ± 0.02^a^, 100.00	48.11 ± 0.19^b^, 97.94	22.96 ± 0.27^a^, 99.40	98.53	VH
IS8123	Brown	329.91 ± 4.42^e^, 75.98	5.69 ± 0.05^efg^, 54.83	28.52 ± 0.03^b^, 96.76	43.72 ± 0.26^d^, 89.00	19.55 ± 0.35^d^, 84.63	80.24	VH
IS8023	Orange	115.07 ± 1.39^j^, 26.50	3.45 ± 0.03^k^, 33.25	13.12 ± 0.11^j^, 44.52	21.38 ± 0.20^k^, 43.52	7.62 ± 0.19l^m^, 33.00	36.16	L
IS8044	White	22.32 ± 0.70^m^, 5.14	1.14 ± 0.01^o^, 11.03	0.71 ± 0.01^o^, 2.42	1.53 ± 0.02^r^, 3.11	0.48 ± 0.01^r^, 2.09	4.76	VL
IS8017	Orange	305.43 ± 6.62^f^, 70.34	5.86 ± 0.04^ef^, 56.50	28.84 ± 0.07^ab^, 97.88	46.81 ± 0.02^c^, 95.29	21.97 ± 0.64^b^, 95.13	83.03	VH
IS8127	Yellow	161.52 ± 1.73^i^, 37.20	3.82 ± 0.01^j^, 36.77	14.36 ± 0.05^i^, 48.73	23.15 ± 0.03^j^, 47.12	8.30 ± 0.19^l^, 35.93	41.15	M
ET 185–2	White	0.68 ± 0.00^n^, 0.16	1.00 ± 0.01^o^, 9.66	0.24 ± 0.00^o^, 0.81	1.42 ± 0.01^r^, 2.88	0.56 ± 0.01^r^, 2.40	3.18	VL
ET 36–1	White	1.28 ± 0.00^n^, 0.29	0.93 ± 0.00^o^, 8.94	0.24 ± 0.00^o^, 0.80	1.31 ± 0.01^r^, 2.66	0.46 ± 0.01^r^, 1.97	2.93	VL
IS 12611	White	1.28 ± 0.00^n^, 0.29	0.92 ± 0.01^o^, 8.87	0.24 ± 0.00^o^, 0.80	1.22 ± 0.00^r^, 2.48	0.40 ± 0.01^r^, 1.72	2.83	VL
Setokou 1	Yellow	72.95 ± 4.08^k^, 16.80	3.61 ± 0.22^jk^, 34.79	3.54 ± 0.21^m^, 12.01	9.43 ± 0.23^o^, 19.20	3.04 ± 0.11^o^, 13.18	19.20	VL
JN 36	White	26.32 ± 1.27^lm^, 6.06	2.24 ± 0.03^m^, 21.57	3.19 ± 0.12^mn^, 10.81	1.25 ± 0.04^r^, 2.54	0.94 ± 0.07^qr^, 4.07	9.01	VL
JN 69	White	62.97 ± 3.15^k^, 14.50	1.84 ± 0.00^n^, 17.73	0.97 ± 0.04^o^, 3.29	1.32 ± 0.05^r^, 2.69	0.68 ± 0.01^qr^, 2.96	8.24	VL
Gangwonsusu 6	Orange	244.27 ± 1.71^g^, 56.25	5.96 ± 0.07^e^, 57.39	16.35 ± 0.52^h^, 55.49	19.18 ± 0.14^l^, 39.04	10.50 ± 0.17^k^, 45.47	50.73	M
Nampungchal^*^	Orange	323.36 ± 0.73^e^, 74.47	6.31 ± 0.03^d^, 60.79	18.79 ± 0.17^f^, 63.75	39.85 ± 0.42^e^, 81.13	13.72 ± 0.35^h^, 59.40	67.91	H
Wheatland^*^	Orange	58.61 ± 2.15^k^, 13.50	1.82 ± 0.14^n^, 17.53	1.05 ± 0.05^o^, 3.57	6.14 ± 0.31^p^, 12.49	1.90 ± 0.07^p^, 8.22	11.06	VL
Sodamchal^*^	Orange	241.15 ± 3.30^gh^, 55.54	5.74 ± 0.07^ef^, 55.28	17.57 ± 0.33^g^, 59.62	36.79 ± 0.17^f^, 74.89	12.41 ± 0.33^i^, 53.73	59.81	M
Total range		0.68–434.22	0.92–10.38	0.24–49.12	1.22–49.19	0.40–23.10	2.83–98.53	
Total mean		186.01	4.51	14.11	23.66	9.96	45.09	
CV (%)		74.69	59.99	75.41	75.56	80.56	71.59	

Values represent means ± standard deviations (*n* = 3) analyzed in triplicates. Mean values in a column marked by different superscript letters are significantly different (*p* < 0.05). ABTS, ABTS^•+^ scavenging activity; AI, Antioxidant index; CV, Coefficient of variation; DPPH, DPPH-radical scavenging activity; FRAP, Ferric reducing antioxidant power; H, High; L: Low; M, Medium; TPC, Total phenolic content; TTC, Total tannin content; VH, Very high; VL, Very low; ^*^Cultivar.

The scattergrams in Supplementary Figure S2 illustrate the distribution of the sorghum resources based on genotype. In the cultivars, TTC, TPC, and each of the antioxidant activities decreased in the order of Nampungchal > Sodamchal > Wheatland. In comparison to the three cultivars, 19.23, 17.39, 43.48, 30.43, and 39.13% of the breeding lines had higher TTC, TPC, DPPH^•^ scavenging activity, ABTS^•+^ scavenging activity, and FRAP, respectively. Among them, three breeding lines including IS8009, IS8107, and IS8121 exhibited significantly higher values of each trait (*p* < 0.05). On the other hand, ET36-1, ET185-2, and IS12611 had significantly lower TTC, TPC, FRAP, and ABTS^•+^ scavenging activity than all of the cultivars (Table 1). High levels of TPC and TTC are desirable parameters in sorghum seeds owing to their health benefits as highlighted before (39, 40). Therefore, those breeding lines with high levels of TPC and TTC could be good sources of antioxidants. Contrary to their benefits as antioxidants, polyphenols, particularly tannins, tend to form complexes with dietary protein, minerals, and carbohydrates, slowing their digestion and absorption. Therefore, low and/or non-tannin sorghum varieties are also desired in food industries and are among the primary breeding objectives (1, 41). Our study found a much lower TTC level (< 1.50 mg CE/g) in three breading lines including ET 185–2 (0.68 mg CE/g), ET 36–1 (1.28 mg CE/g), and IS12611 (1.28 mg CE/g) and hence, they could be important resources.

### 3.2. Antioxidant index

AI is an inclusive method for ranking food products and plant genetic materials based on their overall antioxidant capacity (32, 42, 43). The AI of each sorghum was also evaluated, and a wide-ranging AI value was found among the sorghum genotypes. Accordingly, six genotypes were classified as very high (80–100%), four as high (60–79%), four as medium (40–59%), three as low (20–39%), and nine as very low (0–19%) AI (Table 1). As expected, genotypes with higher levels of TPC and TTC had a higher AI value, and vice versa. Individually, all seven genotypes that had low or very low AI values were white sorghums showing the potential relationship between seed color and AI parameters. Except for IS8127 and Setokou 1, which had medium and very low AI values, respectively, genotypes with yellow seed colors had either high or very high AI values. In orange genotypes, the AI ranged from very low to very high. The only red and brown genotypes, Darset and IS8123, were classified as low and very high AI genotypes, respectively. Apart from their biological activities, these findings support the role of tannins in the pigmentation of the testa and pericarp, which influences sorghum seed color (44). The differences in AI values also prompted us to group the sorghum genotypes based on seed color and statistically investigate the variance of each metabolite examined, as will be explained in detail in a separate section.

### 3.3. Total protein, total Fat, and fiber contents

Table 2 shows the total protein, total fat, total dietary fiber, and total crude fiber contents of each sorghum genotype, and each of the parameters varied significantly (*p* < 0.05). Total protein and total fat levels were in the ranges of 9.05–14.61 and 2.99–6.91%, with mean values of 11.58 and 4.24%, respectively. Similarly, total dietary fiber and total crude fiber contents were in the ranges of 6.72–16.27 and 0.71–2.62%, with means of 10.51 and 1.51%, respectively. Compared to our study, Htet et al. (45) found comparable fat (2.89–5.14%) and crude fiber (1.58–2.49%) contents in six sorghum varieties grown in China. Protein and lipid levels of 9.57–11.13 and 3.60–5.43%, respectively, have also been reported in five Brazilian sorghum hybrids (16). Another study compared the fat contents of sorghum varieties with other cereal grains and found a total fat content of 3.77% in red and 3.61% in white sorghums (46). The same study reported total protein contents of 6.24 and 9.34%. Other studies evaluated the nutritional qualities of individual and/or groups of sorghum genotypes under different treatment conditions (47–50). Again, factors such as genetic variation, analysis techniques, and growth conditions could all contribute to the observed wide range of values (16, 23, 51).

**Table 2:** Nutritional contents in the seeds of sorghum genotypes on a dry seed weight basis.

Genotype	Total protein (%)	Total fat (%)	Total dietary fiber (%)	Total crude fiber (%)
Bonita	10.34 ± 0.02l^m^	3.26 ± 0.14^jk^	8.32 ± 0.25^ij^	1.41 ± 0.04^gh^
Darset	12.68 ± 0.09^e^	3.90 ± 0.04^gh^	12.64 ± 0.80^c^	1.28 ± 0.04^ij^
IS8005	11.96 ± 0.01^h^	5.27 ± 0.15^d^	10.42 ± 0.77^fg^	1.22 ± 0.04^j^
IS8009	13.21 ± 0.12^c^	6.91 ± 1.12^a^	14.45 ± 1.05^b^	1.56 ± 1.09^ef^
IS8014	9.51 ± 0.05^o^	6.32 ± 0.11^b^	13.05 ± 0.77^c^	1.38 ± 0.04^hi^
IS8051	10.29 ± 0.05^m^	5.67 ± 0.29^c^	10.31 ± 0.48^fgh^	1.30 ± 0.04^ij^
IS8089	9.05 ± 0.04^q^	3.27 ± 0.18^jk^	7.96 ± 0.32^ij^	0.97 ± 0.02^k^
IS8093	10.44 ± 0.07^l^	2.99 ± 0.11^k^	9.09 ± 0.53^hi^	1.50 ± 0.08^fg^
IS8107	12.43 ± 0.02^f^	3.02 ± 0.12^k^	10.60 ± 0.34^ef^	2.23 ± 0.08^b^
IS8112	11.63 ± 0.07^i^	3.00 ± 0.24^k^	11.92 ± 0.51^cd^	2.28 ± 0.08^b^
IS8121	10.45 ± 0.06^l^	3.12 ± 0.09^k^	9.81 ± 0.29^fgh^	1.63 ± 0.03^e^
IS8123	11.64 ± 0.05^i^	3.58 ± 0.16^hi^	11.74 ± 0.45^cde^	1.64 ± 0.09^e^
IS8023	12.30 ± 0.03^f^	4.48 ± 0.05^ef^	16.27 ± 0.65^a^	1.97 ± 0.04^c^
IS8044	13.55 ± 0.08^b^	6.30 ± 0.07^b^	11.92 ± 0.36^cd^	2.62 ± 0.11^a^
IS8017	10.72 ± 0.12^k^	3.96 ± 0.08^g^	7.96 ± 0.66^ij^	1.05 ± 0.01^k^
IS8127	9.28 ± 0.03^p^	3.89 ± 0.13^gh^	12.03 ± 0.64^cd^	1.98 ± 0.06^c^
ET 185–2	12.08 ± 0.08^g^	3.75 ± 0.12^ghi^	9.65 ± 0.49^fgh^	2.08 ± 0.00^c^
ET 36–1	12.66 ± 0.04^e^	3.74 ± 0.03^ghi^	9.27 ± 0.38^ghi^	1.80 ± 0.05^d^
IS 12611	12.30 ± 0.03^f^	3.55 ± 0.25^ij^	9.18 ± 0.23^ghi^	1.62 ± 0.04^ef^
Setokou 1	12.84 ± 0.05^d^	3.64 ± 0.14^hi^	12.25 ± 0.84^c^	1.54 ± 0.05^ef^
JN 36	13.10 ± 0.05^c^	4.69 ± 0.21^e^	12.47 ± 0.32^c^	1.27 ± 0.03^ij^
JN 69	11.11 ± 0.06^j^	5.65 ± 0.05^c^	8.11 ± 0.66^fij^	0.86 ± 0.04^l^
Gangwonsusu 6	10.74 ± 0.02^k^	4.70 ± 0.07^e^	10.46 ± 0.36^fg^	1.05 ± 0.03^k^
Nampungchal^*^	9.82 ± 0.01^n^	4.38 ± 0.07^f^	9.01 ± 0.20^hij^	0.71 ± 0.06^m^
Wheatland^*^	14.61 ± 0.06^a^	3.45 ± 0.07^ij^	6.72 ± 0.70^k^	1.25 ± 0.00^j^
Sodamchal^*^	12.37 ± 0.06^f^	3.76 ± 0.07^ghi^	7.73 ± 0.35^jk^	1.04 ± 0.02^k^
Total range	9.05–14.61	2.99–6.91	6.72–16.27	0.71–2.62
Total mean	11.58	4.24	10.51	1.51
CV (%)	12.13	26.14	21.18	30.65

Values represent means ± standard deviations (*n* = 3) analyzed in triplicates. Mean values in a column marked by different superscript letters are significantly different (*p* < 0.05).

^*^Cultivar.

Once again, Supplementay Figure S2 displays the genotype-specific distribution of sorghum resources across nutritional factors. Accordingly, none of the breeding lines had a higher total protein content than Wheatland. However, 30.43% had a greater total protein content than the other two cultivars, with five breeding lines (Darset, IS8009, IS8044, ET 36–1, Setokou 1, JN 36) having a significantly higher value (*p* < 0.05). In terms of fat content, 39.13% had a higher value than all the cultivars, with eight breeding lines (IS8005, IS8009, IS8014, IS8051, IS8044, JN 36, JN 69, and Gangwonsusu 6) having a significantly higher total fat content (Table 2). Protein and fat are among the metabolites that have helped sorghum gain popularity in the food and biofuel industries. In this regard, breeding lines with high levels of fat and protein may be good sources of nutrition. Moreover, they could be promising candidates for developing sorghum varieties with optimal nutritional values (52). Compared to the cultivars, 78.26 and 82.61% of the landraces had higher levels of crude fiber content and dietary fiber content, respectively. Only four breeding lines including JN 69, Gangwonsusu 6, IS8017, and IS8089, had significantly lower crude fiber content than Wheatland (1.25%). Among these, only the former had a significantly lower crude fiber content than Sodamchal (1.04%). Nampungchal has the lowest crude fiber content (0.71%) of all the sorghum genotypes. Similarly, the majority of the breeding lines showed a significantly higher dietary fiber level than the cultivars (*p* < 0.05). Apart from their numerous health benefits, fibers play important roles in improving the quality of gluten-free dietary products and are thus important components of sorghum seeds (17, 51). Accordingly, most of the breeding lines could be potential sources of high fiber levels.

### 3.4. Individual and total fatty acid contents

**Figure 1:**
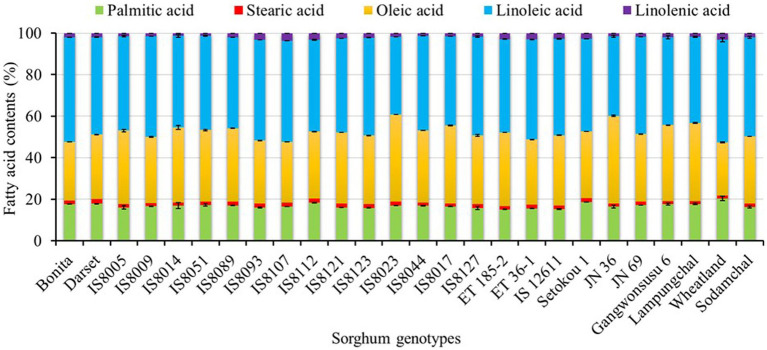
Stacked bar plot representing the relative variations of fatty acid contents in seeds of sorghum genotypes.

Despite their relatively low levels of fat content, sorghum seeds are known for their fatty acid compositions, which play crucial roles in disease prevention (53). Our GC-FID analysis found two saturated fatty acids (palmitic acid and stearic acid) and three unsaturated fatty acids (oleic acid, linoleic acid, and linolenic acid) in all the breeding lines and cultivars with significantly varied contents (p < 0.05). The stack bar chart in Figure 1 depicts the levels of each fatty acid in the sorghum genotypes. The numerical values and statistical data are provided in Supplementary Table S1. Palmitic acid content ranged from 15.15 to 20.39%, whereas stearic acid content ranged from 1.30 to 2.11%. Oleic acid was the only monounsaturated fatty acid found, with contents ranging from 25.62 to 42.31%. The contents of linoleic and linolenic acid, the two polyunsaturated fatty acids, were in the ranges of 37.47–50.39 and 1.22–3.75%, respectively. Several studies reported comparable fatty acid contents in several sorghum resources. Ryu et al. (47), for example, reported a palmitic acid content of 18.63–29.89%, stearic acid content of 0.00–1.62%, oleic acid content of 14.01–31.68%, linoleic acid content of 40.86–47.07, and linolenic acid content of 3.90–11.17% in Korean sorghum cultivars. Liu et al. (46) also compared the fatty acids of two sorghum resources with other cereal grains and discovered 16.83 and 17.21% palmitic acid, 2.44 and 1.85% stearic acid, 36.64 and 31.60% oleic acid, 40.10 and 45.44% linoleic acid, and 2.27 and 2.16% linolenic acid. Palmitic acid was found to be the most abundant saturated fatty acid in our study, which agreed with many of the previous studies. Similarly, linoleic acid was found to be the most dominant unsaturated fatty acid except in two breeding lines, IS8023 and JN 36, where oleic acid predominated. Such exceptions have previously been observed, indicating the effect of genetic variation on fatty acid levels (6).

The relative distribution of sorghum resources in terms of fatty acid levels by genotype was also investigated (Supplementary Figure S2). Wheatland had the highest palmitic acid, the lowest oleic acid, the second-highest linoleic acid, and the third-highest linolenic acid contents (*p* < 0.05). The majority of the breeding lines (65.21%) exhibited a higher stearic acid content when compared to cultivars. In contrast, only three breeding lines including IS8017, IS8023, and JN 36 displayed a higher oleic acid level than all the cultivars. Similarly, only Bonita had a significantly higher linoleic acid content than all the cultivars, while IS8112 and IS8107 had a higher linolenic acid content. In general, total polyunsaturated fatty acid (PUFA) content (39.20–52.52%) was higher than total monounsaturated content (MUFA) (25.62–42.31%) and total saturated fatty acid (SFA) content (16.82–21.86%) in all genotypes except those two breeding lines (IS8023 and JN 36). A higher ratio of unsaturated to saturated fatty acids has been linked to reducing the risk of cardiovascular diseases (53). Therefore, genotypes such as Bonita, IS8051, IS8089, IS8127, and Gangwonsusu 6 which displayed significantly high levels of PUFA could be ideal resources. Another interesting observation in this study was that genotypes with a high oleic acid content tend to contain lower levels of linoleic and linolenic acid. Numerous fatty acid desaturase enzymes catalyze the interconversion of oleic acid to linoleic acid and linolenic acid in sorghum, as well as in many other crops. As a result, such inverse levels are commonly anticipated (54).

### 3.5. 3-deoxyanthocyanidins and flavonoid contents

Comparatively to the leaf sheath and bran, the levels of anthocyanins and flavonoids in sorghum seeds are rarely studied. Moreover, there are not many studies that consider a large population of sorghum genotypes (10, 12, 55). The UPLC-MS/MS analysis in our study found variations in the distribution and concentration of the targeted 3-deoxyanthocyanidins and flavonoids (Table 3). Except for eriodictyol, a flavanone biomolecule, all of the target molecules were detected in the control cultivars, however, there was variation in the breeding lines. None of the 3-deoxyanthocyanidins were found in ET185-2 and IS12611. Similarly, 5-methoxyluteolinidin was not detected in IS8005, IS8009, and IS8014, whereas luteolinidin and apigeninidin were not found in IS8005 and ET36-1. 7-methoxyapigeninidin was not found in IS8005 and ET36-1. In terms of flavonoids, apigenin was not detected in Darset, IS8121, ET36-1, and JN-69, while luteolin was detected in all other sorghum genotypes except IS8005. Eriodictyol was not found in any of the sorghum breeding lines. Such variations in 3-deoxyanthocyanidins and flavonoid distribution have previously been noted, and they might be caused by variances in the genetic makeup and growth circumstances (9, 56).

**Table 3:** Distribution and contents of individual flavonoids and anthocyanin in sorghum genotypes.

Genotype	Luteolinidin (mg/g)	Apigeninidin (mg/g)	5-MeLu (mg/g)	7-MeAp (mg/g)	Luteolin (mg/g)	Apigenin (mg/g)
Bonita	1.33 ± 0.08^c^	0.57 ± 0.03^m-n^	1.41 ± 0.07^c^	0.51 ± 0.01^d-g^	0.15 ± 0.01^j-k^	0.02 ± 0.00^g^
Darset	0.22 ± 0.04^i-k^	0.17 ± 0.02^p^	0.35 ± 0.07^h-j^	0.17 ± 0.01^g^	0.03 ± 0.01^m^	ND
IS8005	ND	0.23 ± 0.03^p^	ND	ND	ND	0.55 ± 0.13^c-d^
IS8009	0.16 ± 0.00^j-k^	0.98 ± 0.01^j^	ND	0.24 ± 0.00^f-g^	0.16 ± 0.00^j-k^	0.60 ± 0.01^c^
IS8014	0.15 ± 0.00^k^	1.16 ± 0.01^h-i^	ND	0.21 ± 0.00^g^	0.14 ± 0.00^k^	0.75 ± 0.01^b^
IS8051	0.16 ± 0.00^j-k^	1.08 ± 0.03^i^	0.13 ± 0.00^l^	0.29 ± 0.01^f-g^	0.15 ± 0.00^k^	0.79 ± 0.05^b^
IS8089	1.32 ± 0.06^c^	2.80 ± 0.08^b^	1.15 ± 0.02^d^	1.62 ± 0.04^b^	0.43 ± 0.02^e^	0.18 ± 0.01^e-f^
IS8093	0.39 ± 0.02^g-h^	1.93 ± 0.06^d^	0.29 ± 0.01^jk^	1.06 ± 0.06^c^	0.27 ± 0.00^h^	0.15 ± 0.00^e-g^
IS8107	0.42 ± 0.01^f-g^	0.59 ± 0.01^m-n^	0.46 ± 0.01^h^	0.36 ± 0.01^f-g^	0.26 ± 0.01^h^	0.03 ± 0.00^g^
IS8112	0.31 ± 0.02^g-i^	1.31 ± 0.03^g^	0.23 ± 0.01^j-l^	0.71 ± 0.03^c-g^	0.37 ± 0.00^f^	0.08 ± 0.00^f-g^
IS8121	0.61 ± 0.02^e^	0.24 ± 0.00^p^	0.61 ± 0.03^g^	0.17 ± 0.00^g^	0.18 ± 0.00^i-j^	ND
IS8123	0.54 ± 0.01^e-f^	2.06 ± 0.06^c^	0.43 ± 0.00^h-i^	0.96 ± 0.01^c-e^	0.16 ± 0.00^j-k^	0.09 ± 0.00^e-g^
IS8023	0.76 ± 0.01^d^	0.37 ± 0.01^o^	0.60 ± 0.01^g^	0.23 ± 0.00^f-g^	0.15 ± 0.00^jk^	0.07 ± 0.00^f-g^
IS8044	0.30 ± 0.01^g-j^	1.22 ± 0.02^h^	0.31 ± 0.00^i-j^	1.03 ± 0.01^c-d^	0.19 ± 0.00^i^	0.23 ± 0.01^e^
IS8017	0.53 ± 0.01^e-f^	1.93 ± 0.05^d^	0.15 ± 0.00^k-l^	0.77 ± 0.03^c-f^	1.67 ± 0.02^a^	6.35 ± 0.23^a^
IS8127	1.99 ± 0.05^b^	0.75 ± 0.01^l^	1.26 ± 0.03^d^	0.38 ± 0.01^f-g^	0.33 ± 0.01^g^	0.03 ± 0.00^g^
ET 185–2	ND	ND	ND	ND	0.15 ± 0.00^k^	0.46 ± 0.01^d^
ET 36–1	0.22 ± 0.00^i-k^	ND	0.16 ± 0.00^kl^	ND	0.15 ± 0.00^j-k^	ND
IS 12611	ND	ND	ND	ND	0.09 ± 0.00^l^	0.13 ± 0.01^e-g^
Setokou 1	0.67 ± 0.01^d-e^	1.71 ± 0.04^e^	0.92 ± 0.00^e^	1.16 ± 0.01^b-c^	0.39 ± 0.00^f^	0.16 ± 0.00^e-g^
JN 36	2.01 ± 0.01^b^	0.52 ± 0.0^1-n^	2.06 ± 0.05^b^	0.46 ± 0.01^e-g^	0.51 ± 0.01^d^	0.04 ± 0.00^f-g^
JN 69	3.67 ± 0.25^a^	0.64 ± 2.03^m^	2.78 ± 0.22^a^	0.41 ± 0.02^f-g^	0.55 ± 0.04^c^	ND
Gangwonsusu 6	1.47 ± 0.03^c^	7.52 ± 0.07^a^	1.28 ± 0.06^c-d^	3.20 ± 1.07^a^	0.65 ± 0.01^b^	0.48 ± 0.01^cd^
Nampungchal^*^	0.32 ± 0.01^g-i^	1.60 ± 0.04^f^	0.24 ± 0.01^j-l^	0.71 ± 0.02^c-g^	0.14 ± 0.00^k^	0.09 ± 0.00^e-g^
Wheatland^*^	1.37 ± 0.10^c^	0.85 ± 1.05^k^	0.77 ± 0.07^f^	0.31 ± 0.06^f-g^	0.16 ± 0.02^j-k^	0.03 ± 0.01^g^
Sodamchal^*^	0.25 ± 0.01^h-k^	1.36 ± 0.03^g^	0.25 ± 0.01^j-l^	0.79 ± 0.17^c-f^	0.16 ± 0.01^i-k^	0.07 ± 0.00^f-g^
Total range	0.15–3.67	0.17–7.52	0.09–2.78	0.17–3.20	0.03–1.67	0.02–6.35
Total mean	0.83	1.38	0.75	0.72	0.32	0.54
CV (%)	99.60	103.28	90.05	92.17	99.31	251.21

Values represent means ± standard deviations (*n* = 3). Mean values in a column marked by different superscript letters are significantly different (*p* < 0.05). CV, Coefficient of variation; 5-MeLu, 5-Methoxyluteolinidin; 7-MeAp, 7-Methoxyapigeninidin; ND, Not detected.

^*^Cultivar.

The levels of the target 3-deoxyanthocyanidins and flavonoids were determined using an optimized UPLC as described before, and significant variations (*p* < 0.05) were observed among the sorghum genotypes (Table 3). With means of 0.83, 1.38, 0.75, and 0.72 mg/g, the concentrations of luteolinidin, apigeninidin, 5-methoxyluteolinidin, 7-methoxyapigeninidin were in the ranges of 0.15–3.68, 0.17–7.52, 0.09–2.06, and 0.17–3.20 mg/g, respectively (p < 0.05). Likewise, luteolin and apigenin contents were in the ranges of 0.03–1.67 and 0.02–6.35 mg/g, respectively with means of 0.32 and 0.54 mg/g. Different groups of researchers reported widely varied contents of 3-deoxyanthocyanidins and flavonoids in several sorghum resources. For example, luteolin and apigenin contents of 3.8–74.0 μg/g and 0.8–287.3 μg/g, respectively have been reported (9). In a similar study, the total 3-deoxyanthocyanidin content ranged from 0.6–186.9 μg/g. A study by Yang et al. (57) found luteolinidin, apigeninidin, and 7-methoxyapigeninidin levels as high as 12.3, 10.1, and 9.4 mg/g, respectively. Other studies have also reported such a wide range of values (10, 56). Despite the differences in extraction and analysis methods, genotype, and growth conditions, the values found in our study are within previously reported ranges. It was notable that genotypes with a high amount of luteolinidin and apigeninidin also had greater levels of their respective methoxy derivatives, which could be related to their shared biosynthetic pathways (58).

Supplementary Figure S2 also depicts the genotype-specific distribution of sorghum resources in terms of 3-deoxyanthocyanidin and flavonoid concentration. Among the cultivars, Wheatland had the highest concentrations of luteolinidin (1.37 mg/g) and 5-methoxyluteolinidin (0.77 mg/g), but the lowest concentration of apigeninidin (0.85 mg/g). Three breeding lines including JN 69, JN 36, and IS8127 had a significantly high level of luteolinidin (3.67, 2.01, and 1.99 mg/g, respectively) than all the cultivars. Likewise, seven breeding lines showed a significantly higher level of 5-methoxyluteolinidin than the three cultivars, whereas six breeding lines had a significantly higher level of apigeninidin (Table 3). In contrast, only two breeding lines, Gangwonsusu 6 and IS8089, displayed a significantly higher level of 7-methoxyapigeninidin (3.20 and 1.62 mg/g, respectively) than the three cultivars. Regarding flavonoid content, the majority of breeding lines outweighed the cultivars. Compared to all the cultivars, only IS12611 and Darset displayed a significantly lower level of luteolin (0.03 and 0.09 mg/g, respectively), while only Bonita exhibited a significantly lower level of apigenin (0.02 mg/g). In a typical way, IS8017 simultaneously displayed the highest levels of both luteolin (1.67 mg/g) and apigenin (6.35 mg/g). High concentrations of 3-deoxyanthocyanidins and flavonoids in sorghum seeds have attracted attention for their pronounced health benefits and disease protection, as well as for their potential use as food colorants (4, 9, 14, 27). The breeding lines with high 3-deoxyanthocyanidins contents, particularly Gangwonsususu 6, JN69, JN 36, IS8089, and IS8123, may therefore be valuable resources.

### 3.6. Effect of seed color

Sorghum seeds, which are controlled by a number of loci, come in a variety of colors, including white, red, yellow, brown, orange, and black, among others, and may alter the levels of metabolites (3, 35, 36). In our study, we observed all the white sorghums to have a very low or low AI value, while the majority of the yellow genotypes have very high or high AI values as described before. To support the observation with statistical data, ANOVA was conducted on genotypes grouped according to seed color (Supplementary Table S2). The result showed significantly low levels of TTC, TPC, and antioxidant capacities in white than yellow and orange genotypes. Previous studies also found significantly low levels of TTC, TPC, and antioxidant activity in white sorghums which agreed with our observation (17, 39). Likewise, a comparable analysis was conducted on the nutritional qualities and fatty acids. However, none of the metabolites were significantly affected by seed color variation. Regarding 3-deoxyanthocyanidins and flavonoids, orange sorghum seeds had a higher average apigeninidin content (2.19 mg/g), while white sorghum seeds had the highest average levels of luteolinidin (1.50 mg/g) and 5-methoxyluteolinidin (1.35 mg/g). Once again, the differences between the various colors were not significantly different (*p* < 0.05). The variations of apigenin and luteolin were likewise not significantly varied between white, yellow, and orange genotypes (Supplementary Table S2). Previously, several studies looked into how seed color could affect the levels of the different classes of sorghum metabolites. However, inconsistent results were reported since the majority of studies concentrated on individual genotypes rather than taking into account a huge population of sorghum resources of various colors (14, 17, 55). Our findings suggest that it is not viable to assess the nutritional values, fatty acid levels, and 3-deoxyanthocyanidin and flavonoid contents of sorghum seeds entirely based on their color, and hence, individual genotypes should be investigated (51, 52). Furthermore, future studies should involve a broad population of sorghum genetic material to make a conclusive statement about the effect of seed color on the levels of these metabolites. Additionally, molecular-level analysis ought to support the results that have so far been observed (10, 17, 39, 55, 56).

### 3.7. Principal component and correlation analysis

**Figure 2:**
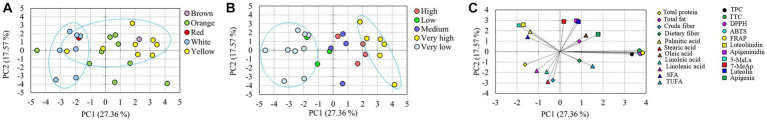
Score plot of sorghum genotypes according to seed color **(A)** and antioxidant index **(B)**, loading plot of variables **(C)** along the first two principal components of the PCA and hierarchical cluster analysis **(D)**. 5-MeLu, 5-Methoxyluteolinidin; 7-MeAp, 7-Methoxyapigeninidin; ABTS, ABTS^•+^ scavenging activity; DPPH, DPPH^•^ scavenging activity; FRAP, Ferric reducing antioxidant power; SFA, Total saturated fatty acid content; TPC, Total phenolic content; TTC, Total tannin content; TUFA, Total unsaturated fatty acid content.

**Figure 3:**
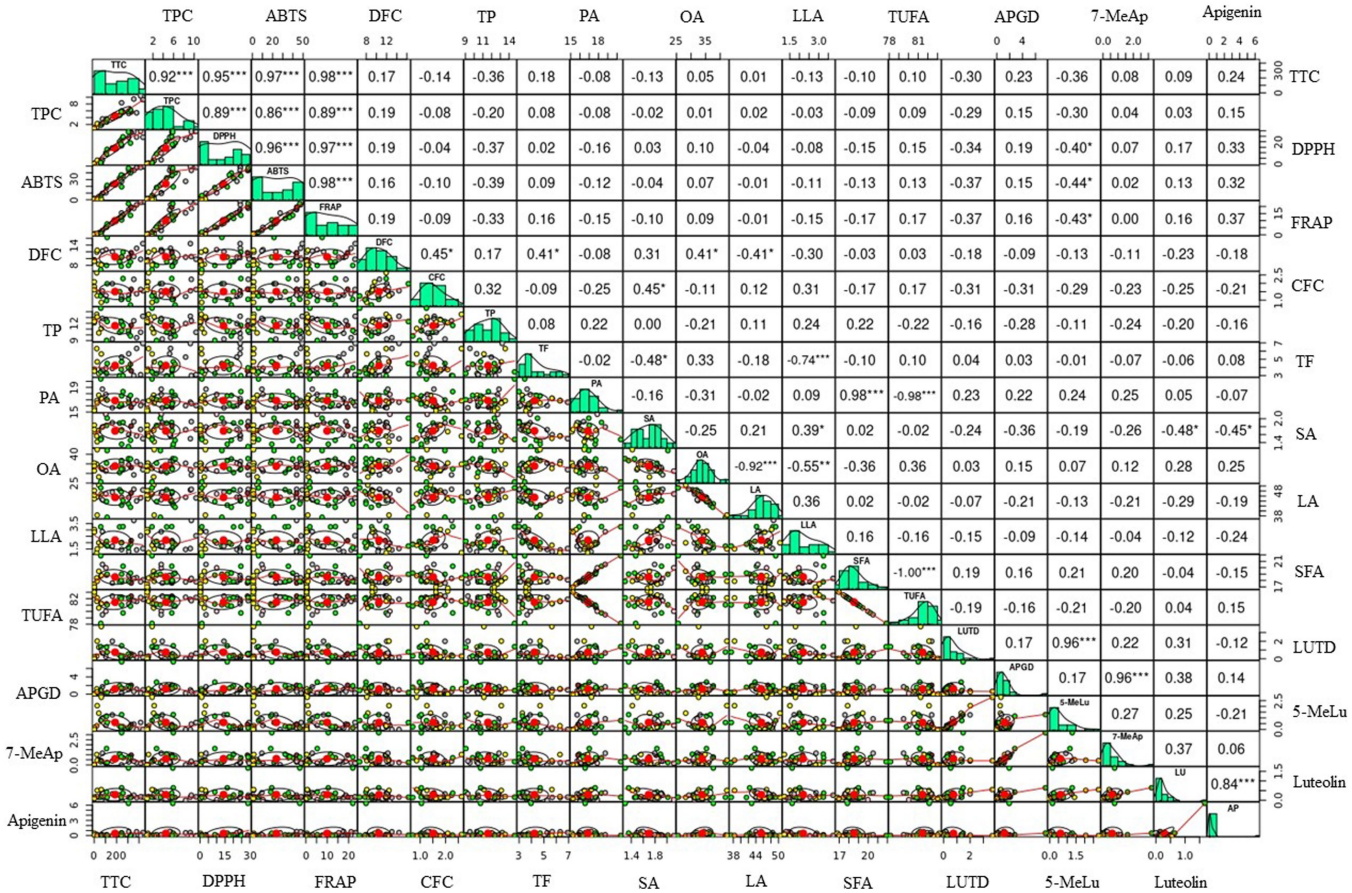
Scatter plots and Pearson’s correlation of metabolites and antioxidant activity from the sorghum genotypes. 5-MeLu, 5-Methoxyluteolinidin; 7-MeAp, 7-Methoxyapigeninidin; ABTS, ABTS^•+^ scavenging activity; AP, Apigenin; APGD, Apigeninidin; CFC, Total crude fiber content; DFC, Total dietary fiber content; DPPH, DPPH^•^ scavenging activity; FRAP, Ferric reducing antioxidant power; LA, Linoleic acid; LLA, Linolenic acid; LU, Luteolin; LUTD, Luteolinidin; OA, Oleic acid; PA, Palmitic acid; SA, stearic acid; SFA, Total saturated fatty acid content; TF, Total fat; TP, Total protein; TTC, Total tannin content; TPC, Total phenolic content; TUFA, Total unsaturated fatty acid content. Correlation coefficient (*r*) values marked by ^*^, ^**^, and ^***^ correspond to significances at *p* < 0.05, *p* < 0.01, and *p* < 0.001, respectively.

Multivariate statistical tools aid in explaining the diversity of plant resources, viewing the interaction of varied metabolites, and evaluating the impact of various factors on metabolite levels. To further understand how the sorghum genotypes are distributed and linked with the measured metabolites and antioxidant activities, PCA and Pearson’s correlation analyzes were computed using the entire data set. PCA yielded six components that had eigenvalues higher than 1 and together accounted for 85.47% of the total variance (Table 4). Out of these, the first two principal components (PC1 and PC2) together accounted for 44.93% of the total variation. Although orange genotypes were widely spread, PCA over PC1 and PC2 tended to cluster the sorghum genotypes according to seed color (Figure 2A). Specifically, white genotypes tend to cluster along the negative side of PC1, while the majority of the yellow genotypes clustered on the positive side. PCA was also computed according to AI values. Interestingly, genotypes with very high AI values clustered on the right extreme along PC1, while genotypes with very low AI values clustered on the left (Figure 2B). As shown in Table 4, the major contributors to the observed variances along PC1 were TTC, TPC, DPPH^•^ scavenging activity, ABTS^•+^ scavenging activity, and FRAP. These traits were also shown to be closely related in the loading plot (Figure 2C). Flavonoids and 3-deoxyanthocyanidins, as well as crude fiber content, were the primary contributors to the variance observed along PC2. Aside from palmitic acid, the impact of individual fatty acids was also considerable along PC2. The remaining variables, including total fat, total protein and dietary fiber content contributed less along PC1 and PC2. Overall, the PCA results in our study revealed that AI parameters including TTC, TPC, DPPH^•^ scavenging activity, ABTS^•+^ scavenging activity, and FRAP could be useful for categorizing a large population of sorghum resources. Scatter plots and Pearson’s correlation analyzes also supported the findings of the PCA. Some significant relationships were identified between the variables analyzed, as shown in Figure 3. TPC, TTC, and antioxidant activity all demonstrated significant and positive relationships with each other. Specifically, TPC exhibited a strong and positive correlation with all antioxidant activities, including DPPH^•^ scavenging activity (r = 0.89), ABTS^•+^ scavenging activity (r = 0.86), and FRAP (r = 0.89), all of which were significant at *p* < 0.001. Similarly, the associations of TTC to DPPH^•^ scavenging activity (r = 0.95), ABTS^•+^ scavenging activity (r = 0.97), and FRAP (r = 0.98) were strong and significant (*p* < 0.001). The positive and significant correlations of TTC and TPC to each of the antioxidant activities demonstrate the roles of tannins and phenols in regulating ROS and RNS, which corroborate multiple other studies (4, 11, 39). In contrast, TTC, TPC, and antioxidant activities showed negative and/or weak correlations with individual fatty acids which could be the result of their distinct biosynthesis routes (54, 59). TTC also displayed weak and negative associations with total protein (r = −0.36) and total fat (r = 0.18) which could be explained by its anti-nutrient effect (1, 41). On the other hand, the positive and significant correlation between apigenin and luteolin (r = 0.84, *p* < 0. 001), luteolinidin and 5-methoxyluteolinidin (r = 0.96, p < 0. 001), and apigeninidin and 7-methoxyapigeninidin (r = 0.96, *p* < 0. 001) could also be explained by their interrelated biosynthetic pathways (58). Among the fatty acids, the negative and significant correlations of oleic acid to linoleic acid (r = −0.92, *p* < 0.001) and linolenic acid (r = −0.55, *p* < 0.01) were noteworthy further supporting the contradictory levels of these unsaturated fatty acids (54).

**Table 4:** Contributions of variables to the variance observed along principal components with eigenvalues of greater than one.

Variables	Contribution (%)					
	PC1	PC2	PC3	PC4	PC5	PC6
TTC	14.64	0.03	3.26	0.13	1.52	0.42
TPC	12.01	0.41	3.25	0.21	2.03	0.02
DPPH	15.05	0.21	2.15	0.00	0.67	1.00
ABTS	15.08	0.18	2.53	0.00	0.39	0.01
FRAP	15.88	0.08	1.89	0.02	0.11	0.12
DFC	0.90	0.47	2.13	28.77	0.57	3.37
Total fat	0.85	3.43	2.17	12.76	0.04	26.79
CFC	0.10	10.21	2.14	2.59	1.62	4.03
Total protein	2.82	2.03	0.03	5.11	16.02	2.34
Palmitic acid	2.14	2.13	19.78	5.20	1.29	0.01
Stearic acid	0.32	13.62	0.00	0.51	5.82	12.70
Oleic acid	1.53	7.09	10.82	4.84	0.08	8.66
Linoleic acid	0.39	7.64	3.22	8.52	0.61	20.00
Linolenic acid	1.24	8.73	3.67	6.29	0.72	9.88
SFA	2.51	0.66	20.20	5.96	0.51	0.58
TUFA	2.51	0.66	20.20	5.96	0.51	0.58
Luteolinidin	3.25	9.80	0.10	0.99	15.89	0.01
Apigeninidin	0.57	6.74	2.36	0.28	0.33	2.86
5-MeLu	4.10	9.03	0.03	0.36	17.67	0.39
7-MeAp	0.03	10.72	1.06	0.68	14.47	3.31
Luteolin	0.74	11.75	0.04	6.89	9.04	5.95
Apigenin	3.35	5.10	0.02	4.60	24.56	0.30
Eigenvalue	5.75	3.69	3.28	2.26	1.55	1.42
Variability (%)	27.36	17.57	15.60	10.78	7.40	6.76
Cumulative (%)	27.36	44.93	60.53	71.31	78.71	85.47

5-MeLu, 5-Methoxyluteolinidin; 7-MeAp, 7-Methoxyapigeninidin; ABTS, ABTS^•+^ scavenging activity; CFC, Total crude fiber content; CV, Coefficient of variation; DFC, Total dietary fiber content; DPPH, DPPH-radical scavenging activity; FRAP, Ferric reducing antioxidant power; SFA, Total unsaturated fatty acid content; TPC, Total phenolic content; TUFA, Total unsaturated fatty acid content.

## 4. Strengths and limitations

This study has several strengths. The study was conducted on a large population of sorghum genotypes that had recently been cultivated in the same environment. The study investigated the variations of several classes of primary and secondary metabolites all at once, as well as antioxidant properties, using both multivariate and univariate statistical tools. Furthermore, the study assessed the effect of seed color variation on the levels of nutritional components, functional metabolites, and antioxidant activities. This study has also some limitations. Despite the study’s emphasis on sorghum metabolites, some proximate analysis parameters such as ash and moisture contents were not determined. Because the study lacked sorghum genotypes of different colors, only the variation between white, yellow, and orange genotypes was statistically analyzed. Furthermore, the research was limited to the most common 3-deoxyanthocyanidins and flavonoids and hence, future metabolite profiling studies on the sorghum genotypes can be conducted to explore other polyphenols.

## 5. Conclusion

This study examined the variations of several classes of nutritional and non-nutritional metabolites in recently cultivated sorghum genotypes. Antioxidant activity was also similarly assessed using three different assays. All of the variables investigated exhibited significant variance among the sorghum genotypes, demonstrating genetic diversity among them. White sorghums were classified as having a very low antioxidant index genotype because they had lower levels of phenolic content, tannin content, and antioxidant activity than the other colored sorghums. Principal component analysis also revealed that phenolic content, tannin content, and antioxidant activity were the most important factors in distinguishing sorghum genotypes based on seed color and antioxidant index. While there were significant differences between individual genotypes, the difference in seed color had no significant effect on nutritional factors such as total protein, total fat, crude fiber, dietary fiber, and individual fatty acids, as well as functional metabolites such as 3-deoxyanthocyanidins and flavonoids. Correlation analysis also revealed several notable relationships between metabolites and/or antioxidant activities. Compared to the control cultivars, some breeding lines were found to have significantly higher levels of metabolite contents and antioxidant activities. Overall, the findings of this study could serve as a foundation for future nutritional and metabolomics research on sorghum genetic resources. Furthermore, those genotypes with high metabolite levels could be utilized in the food industry as well as to develop enhanced sorghum cultivars.

## Data availability statement

The original contributions presented in the study are included in the article/Supplementary material, further inquiries can be directed to the corresponding author.

## Author contributions

SL: conceptualization, funding acquisition, project administration, and methodology. Y-MC: project administration and methodology. M-JS: supervision and resources. HY: conceptualization and methodology. XW: methodology and resources. YL: conceptualization and resources. JY: project administration. Y-aJ: conceptualization, funding acquisition, and project administration. KTD: methodology, investigation, formal analysis, and writing – review & editing. All authors contributed to the article and approved the submitted version.

## Funding

This work was supported by the Research Program for Agricultural Science and Technology Development (Project No. PJ015827) of the National Institute of Agricultural Sciences, Rural Development Administration (Jeonju, Republic of Korea).

## Conflict of interest

The authors declare that the research was conducted in the absence of any commercial or financial relationships that could be construed as a potential conflict of interest.

## Publisher’s note

All claims expressed in this article are solely those of the authors and do not necessarily represent those of their affiliated organizations, or those of the publisher, the editors and the reviewers. Any product that may be evaluated in this article, or claim that may be made by its manufacturer, is not guaranteed or endorsed by the publisher.
