# Supplementary material for: Exploring the potentials of sorghum genotypes: a comprehensive study on nutritional qualities, functional metabolites, and antioxidant capacities
Source: Front Nutr. 2023 Aug 10;10:1238729. doi: 10.3389/fnut.2023.1238729 (PMC10450220; doi:10.3389/fnut.2023.1238729)
Supplement: Supplementary file 1 [file Data_Sheet_1.doc]

Supplementary Material

**Exploring the Potentials of Sorghum Genotypes: A Comprehensive Study on Nutritional Qualities, Functional Metabolites, and Antioxidant Capacities**

**Sukyeung Lee^1^, Yu-Mi Choi^2^, Myoung-Jae Shin^2^, Hyemyeong Yoon^2^, Xiaohan Wang^2^, Yoonjung Lee^2^, Jungyoon Yi^2^, Young-ah Jeon^2^, and Kebede Taye Desta^2,*^**

^1^International Technology Cooperation Center, Technology Cooperation Bureau, Rural Development Administration, Jeonju 54875, Korea

^1^National Agrobiodiversity Center, National Institute of Agricultural Sciences, Rural Development Administration, Jeonju 54874, Korea

*** Correspondence:**[kebetila@gmail.com](mailto:kebetila@gmail.com) (K.T. Desta)


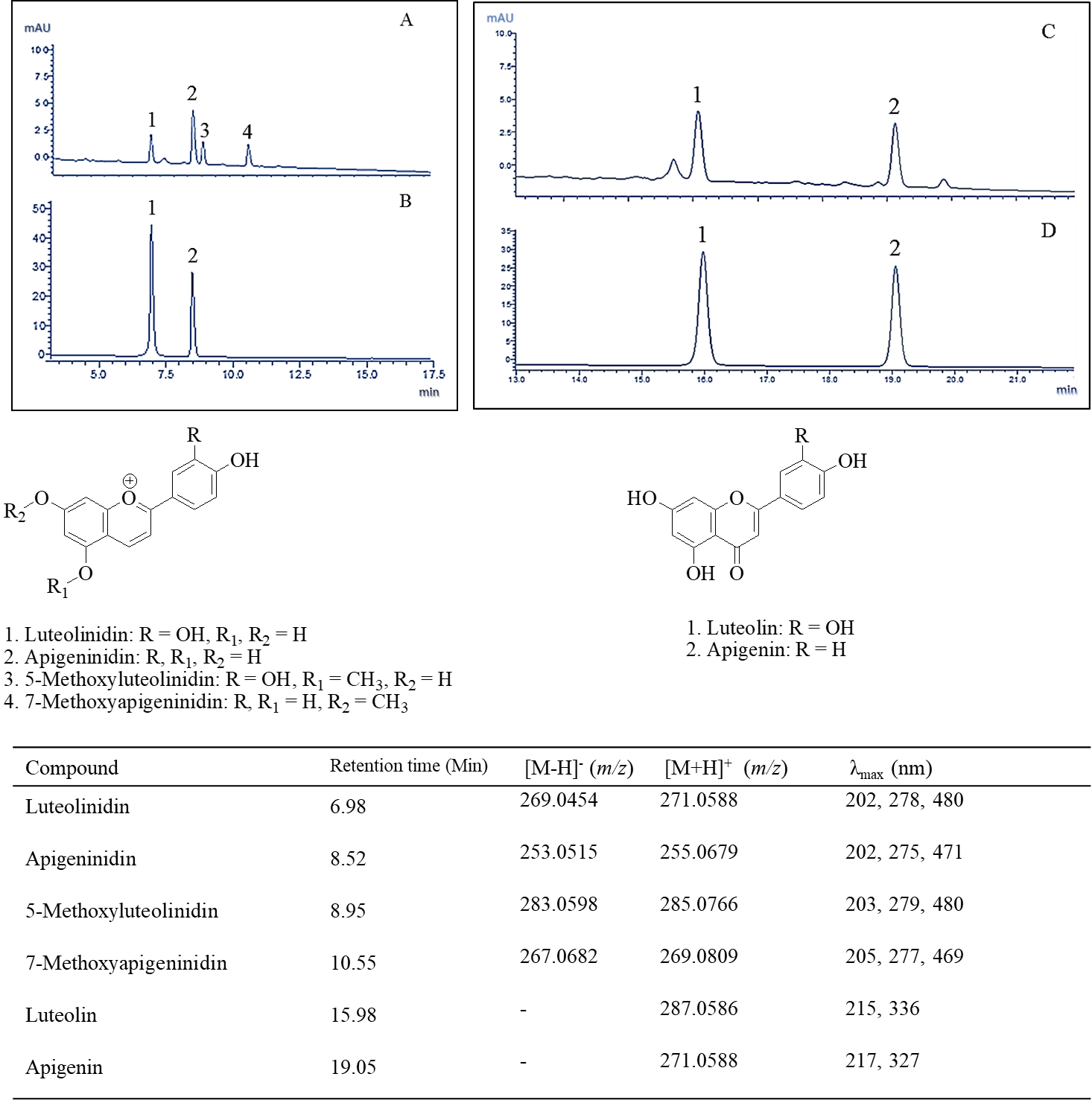


**Scheme S1**. UPLC-MS/MS data for targeted 3-deoxyanthocyanidins (500 nm) and flavonoids (360 nm). LC-chromatograms of sorghum extract (A) and standards (B) for 3-deoxyanthocyanidins, and sorghum extract (C) and standards (D) for flavonoids.


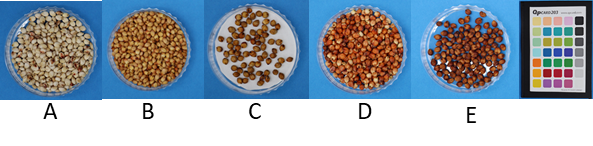


**Figure S1.** Representative sorghum seed samples of different colors. White (A), Yellow (B), Orange (C), Red (D), and Brown (E).


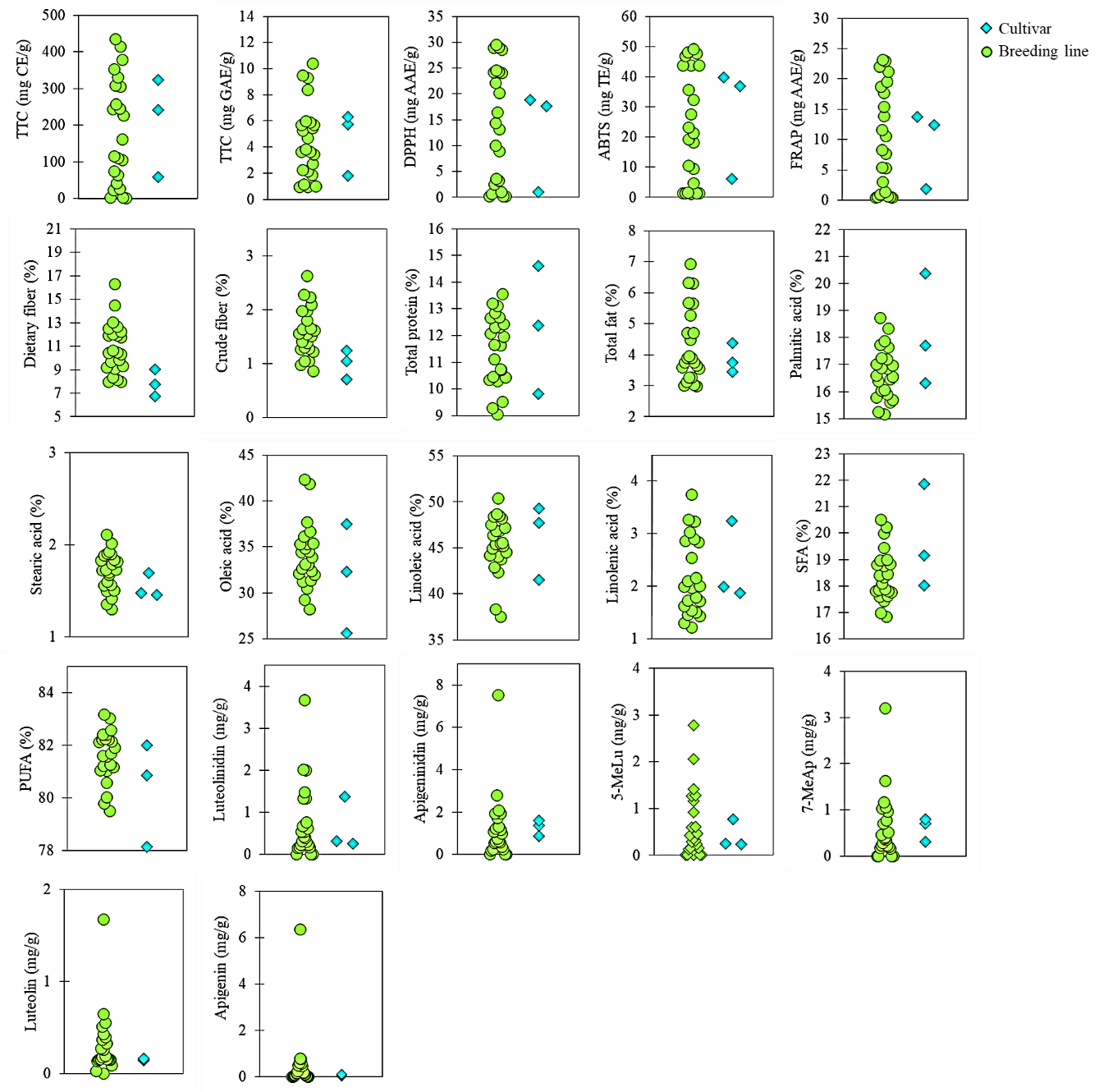


**Figure S2.** Scattergrams showing the distribution of sorghum resources over metabolite contents and antioxidant activities according to genotypes. 5-MeLu: 5-Methoxyluteolinidin; 7-MeAp: 7-Methoxyapigeninidin; ABTS: ABTS**^•^**^+^ scavenging activity; DPPH: DPPH^•^ scavenging activity; FRAP: Ferric reducing antioxidant power; SFA: Total saturated fatty acid content; TPC: Total phenolic content; TTC: Total tannin content; TUFA: Total unsaturated fatty acid content.

**Table S1**. Individual and total fatty acid contents in the seeds of sorghum genotypes.

| Genotype | Palmitic acid  (%) | Stearic acid  (%) | Oleic acid  (%) | Linoleic acid  (%) | Linolenic acid  (%) | SFA (%) | MUFA (%) | PUFA (%) |
| --- | --- | --- | --- | --- | --- | --- | --- | --- |
| Bonita | 17.74 ± 0.10^b-d^ | 1.71 ± 0.03^g-h^ | 28.18 ± 0.09^m^ | 50.39 ± 0.09^a^ | 1.99 ± 0.05^g-h^ | 19.44 ± 0.12^b-e^ | 28.18 ± 0.09^m^ | 52.38 ± 0.07^a^ |
| Darset | 17.87 ± 0.84^b-d^ | 2.11 ± 0.14^a^ | 31.21 ± 0.59^j^ | 46.85 ± 0.41^f-g^ | 1.97 ± 0.04^h^ | 19.98 ± 0.96^b-d^ | 31.21 ± 0.59^j^ | 49.95 ± 0.43^c^ |
| IS8005 | 16.04 ± 0.19^i-m^ | 1.72 ± 0.03^g-h^ | 35.28 ± 0.15^d^ | 45.50 ± 0.07^h^ | 1.46 ± 0.02^m^ | 17.76 ± 0.21^g-k^ | 35.28 ± 0.15^d^ | 45.44 ± 0.22^k^ |
| IS8009 | 16.59 ± 1.45^e-k^ | 1.50 ± 0.04^k-l^ | 31.96 ± 1.06^i^ | 48.41 ± 0.54^c^ | 1.54 ± 0.10^l-m^ | 18.09 ± 1.49^f-j^ | 31.96 ± 1.06^i^ | 46.72 ± 0.02^j^ |
| IS8014 | 16.87 ± 0.60^d-j^ | 1.57 ± 0.02^j-k^ | 36.13 ± 0.39^c^ | 44.01 ± 0.27^j-k^ | 1.43 ± 0.05^m^ | 18.44 ± 0.62^e-i^ | 36.13 ± 0.39^c^ | 45.85 ± 0.04^k^ |
| IS8051 | 17.20 ± 0.10^d-h^ | 1.60 ± 0.00^i-j^ | 34.48 ± 0.09^e^ | 45.42 ± 0.01^h^ | 1.30 ± 0.01^n^ | 18.80 ± 0.11^e-h^ | 34.48 ± 0.09^e^ | 51.74 ± 0.06^b^ |
| IS8089 | 17.00 ± 0.22^d-i^ | 1.83 ± 0.02^d-f^ | 35.31 ± 0.20^d^ | 43.75 ± 0.08^k^ | 2.10 ± 0.05^f-g^ | 18.83 ± 0.24^d-g^ | 35.31 ± 0.20^d^ | 52.43 ± 0.06^a^ |
| IS8093 | 15.91 ± 0.00^i-m^ | 1.89 ± 0.01^c-e^ | 30.47 ± 0.05^k^ | 48.50 ± 0.04^c^ | 3.24 ± 0.05^b^ | 17.80 ± 0.01^g-k^ | 30.47 ± 0.05^k^ | 47.42 ± 0.04^g-h^ |
| IS8107 | 16.39 ± 0.15^g-k^ | 1.93 ± 0.01^c^ | 29.25 ± 0.10^l^ | 48.69 ± 0.20^b-c^ | 3.75 ± 0.14^a^ | 18.32 ± 0.16^e-i^ | 29.25 ± 0.10^l^ | 47.76 ± 0.03^g^ |
| IS8112 | 18.33 ± 0.02^b-c^ | 1.91 ± 0.01^c-d^ | 32.34 ± 0.03^h-i^ | 44.16 ± 0.03^j-k^ | 3.26 ± 0.06^b^ | 20.23 ± 0.02^b-c^ | 32.34 ± 0.03^h-i^ | 49.35 ± 0.03^d-e^ |
| IS8121 | 16.07 ± 0.13^h-m^ | 1.73 ± 0.02^g-h^ | 34.44 ± 0.13^e^ | 45.22 ± 0.05^h^ | 2.54 ± 0.08^e^ | 17.80 ± 0.11^g-k^ | 34.44 ± 0.13^e^ | 39.20 ± 0.03^o^ |
| IS8123 | 15.79 ± 0.01^j-m^ | 1.82 ± 0.00^e-f^ | 33.05 ± 0.03^g^ | 47.19 ± 0.02^e-f^ | 2.16 ± 0.05^f^ | 17.60 ± 0.01^h-k^ | 33.05 ± 0.03^g^ | 46.78 ± 0.01^i-j^ |
| IS8023 | 16.95 ± 0.03^d-i^ | 2.01 ± 0.01^b^ | 41.84 ± 0.01^a^ | 37.47 ± 0.02^p^ | 1.73 ± 0.05^j-k^ | 18.97 ± 0.03^d-g^ | 41.84 ± 0.01^a^ | 44.49 ± 0.30^l^ |
| IS8044 | 16.84 ± 0.14^d-j^ | 1.56 ± 0.01^j-k^ | 34.82 ± 0.16^d-e^ | 45.56 ± 0.01^h^ | 1.22 ± 0.03^n^ | 18.40 ± 0.15^e-i^ | 34.82 ± 0.16^d-e^ | 49.26 ± 0.04^d-e^ |
| IS8017 | 16.56 ± 0.74^f-k^ | 1.30 ± 0.02^n^ | 37.66 ± 0.47^b^ | 42.87 ± 0.38^l-m^ | 1.62 ± 0.09^k-l^ | 17.85 ± 0.76^g-k^ | 37.66 ± 0.47^b^ | 47.80 ± 0.06^g^ |
| IS8127 | 15.70 ± 0.02^k-m^ | 1.90 ± 0.00^c-e^ | 33.14 ± 0.05^g^ | 47.54 ± 0.02^d-e^ | 1.72 ± 0.02^j-k^ | 17.60 ± 0.02^h-k^ | 33.14 ± 0.05^g^ | 51.21 ± 0.18^b^ |
| ET 185-2 | 15.15 ± 0.15^m^ | 1.68 ± 0.03^h-i^ | 35.37 ± 0.11^d^ | 44.91 ± 0.10^h-i^ | 2.89 ± 0.04^d^ | 16.82 ± 0.17^k^ | 35.37 ± 0.11^d^ | 49.18 ± 0.01^d-f^ |
| ET 36-1 | 15.59 ± 0.31^k-m^ | 1.84 ± 0.01^d-f^ | 31.36 ± 0.15^j^ | 48.18 ± 0.26^c-d^ | 3.03 ± 0.08^c^ | 17.43 ± 0.32^i-k^ | 31.36 ± 0.15^j^ | 47.36 ± 0.51^g-i^ |
| IS 12611 | 15.25 ± 0.01^l-m^ | 1.73 ± 0.00^g-h^ | 33.85 ± 0.01^f^ | 46.35 ± 0.01^g^ | 2.83 ± 0.00^d^ | 16.97 ± 0.00^j-k^ | 33.85 ± 0.01^f^ | 39.82 ± 0.04^n^ |
| Setokou 1 | 18.72 ± 0.68^b^ | 1.79 ± 0.13^f-g^ | 32.13 ± 0.28^h-i^ | 44.49 ± 0.54^i-j^ | 2.86 ± 0.05^d^ | 20.52 ± 0.79^b^ | 32.13 ± 0.28^h-i^ | 48.63 ± 0.64^f^ |
| JN 36 | 16.46 ± 0.07^g-k^ | 1.42 ± 0.01^l-m^ | 42.31 ± 0.04^a^ | 38.31 ± 0.04^o^ | 1.50 ± 0.00^l-m^ | 17.88 ± 0.08^g-k^ | 42.31 ± 0.04^a^ | 44.34 ± 0.18^l^ |
| JN 69 | 17.24 ± 0.54^c-g^ | 1.51 ± 0.05^k-l^ | 32.62 ± 0.06^i-j^ | 46.83 ± 0.64^f-g^ | 1.79 ± 0.01^i-j^ | 18.75 ± 0.58^e-h^ | 32.62 ± 0.06^i-j^ | 43.36 ± 0.85^m^ |
| Gangwonsusu 6 | 17.64 ± 0.39^c-f^ | 1.36 ± 0.03^m-n^ | 36.67 ± 0.24^c^ | 42.33 ± 0.28^m^ | 2.00 ± 0.11^g-h^ | 18.99 ± 0.41^d-g^ | 36.67 ± 0.24^c^ | 52.52 ± 0.41^a^ |
| Nampungchal | 17.70 ± 1.00^b-e^ | 1.46 ± 0.09^l^ | 37.48 ± 0.24^b^ | 41.49 ± 0.92^n^ | 1.87 ± 0.08^h-i^ | 19.16 ± 1.08^c-f^ | 37.48 ± 0.24^b^ | 49.71 ± 0.23^c-d^ |
| Wheatland | 20.39 ± 0.45^a^ | 1.48 ± 0.03^l^ | 25.62 ± 0.06^n^ | 49.27 ± 0.45^b^ | 3.25 ± 0.04^b^ | 21.86 ± 0.47^a^ | 25.62 ± 0.06^n^ | 48.81 ± 0.37^e-f^ |
| Sodamchal | 16.32 ± 0.26^g-l^ | 1.70 ± 0.06^h^ | 32.28 ± 0.09^h-i^ | 47.71 ± 0.25^d-e^ | 1.99 ± 0.02^g-h^ | 18.01 ± 0.30^f-k^ | 32.28 ± 0.09^h-i^ | 46.96 ± 0.06^h-j^ |
| Total range | 15.15-20.39 | 1.30-2.11 | 25.62-42.31 | 37.47-50.39 | 1.22-3.75 | 16.82-21.86 | 25.62-42.31 | 39.20-52.52 |
| Total mean | 16.86 | 1.69 | 33.82 | 45.44 | 2.19 | 18.55 | 33.82 | 47.63 |
| CV (%) | 6.80 | 12.11 | 10.75 | 6.78 | 32.12 | 6.09 | 10.75 | 7.14 |

Values represent means ± standard deviations (n =3). Different superscript letters in a column indicate significantly different means (*p* < 0.05). SFA: Total saturated fatty acid content; MUFA: Total monounsaturated fatty acid content; PUFA: Total polyunsaturated fatty acid content.

**Table S2**. Statistical analysis showing the effect of seed color on metabolite contents and antioxidant activities.

| Parameter |  | Seed color | | | | |
| --- | --- | --- | --- | --- | --- | --- |
|  |  | White | Orange | Yellow | Red^*^ | Brown^*^ |
| Total tannin content (mg CE/g) |  | 22.24^b^ | 209.00^a^ | 295.74^a^ | 329.91 | 103.83 |
| Total phenolic content (mg GAE/g) |  | 1.46^b^ | 4.69a^b^ | 6.95^a^ | 5.69 | 3.62 |
| DPPH (mg AAE/g) |  | 1.16^b^ | 16.59^a^ | 21.36^a^ | 28.52 | 9.97 |
| ABTS (mg TE/g) |  | 1.81^b^ | 28.47^a^ | 36.51^a^ | 43.72 | 10.45 |
| FRAP (mg AAE/g) |  | 0.69^b^ | 10.99^a^ | 16.30^a^ | 19.55 | 5.28 |
| Dietary fiber content (%) |  | 9.85^a^ | 9.68^a^ | 11.62^a^ | 11.74 | 12.64 |
| Total fat content (%) |  | 4.42^a^ | 3.78^a^ | 4.73^a^ | 3.58 | 3.90 |
| Crude fiber content (%) |  | 1.67^a^ | 1.31^a^ | 1.60^a^ | 1.64 | 1.28 |
| Total protein content (%) |  | 12.16^a^ | 11.30^a^ | 11.25^a^ | 11.64 | 12.68 |
| Palmitic acid (%) |  | 16.32^a^ | 17.42^a^ | 16.70^a^ | 15.79 | 17.87 |
| Stearic acid (%) |  | 1.63^a^ | 1.66^a^ | 1.72^a^ | 1.82 | 2.11 |
| Oleic acid(%)^†^ |  | 34.07^a^ | 34.41^a^ | 33.35^a^ | 33.05 | 31.21 |
| Linoleic acid (%) |  | 45.79^a^ | 44.17^a^ | 46.16^a^ | 47.19 | 46.85 |
| Linolenic acid (%) |  | 2.18^a^ | 2.34^a^ | 2.07^a^ | 2.16 | 1.97 |
| Total saturated fatty acid (%) |  | 17.96^a^ | 19.08^a^ | 18.41^a^ | 17.60 | 19.98 |
| Total polyunsaturated fatty acid (%) |  | 46.53^a^ | 48.83^a^ | 47.07^a^ | 49.95 | 46.78 |
| Luteolinidin (mg/g) |  | 1.50^a^ | 0.75^a^ | 0.77^a^ | 0.22 | 0.54 |
| Apigeninidin (mg/g) |  | 0.74^a^ | 2.19^a^ | 0.84^a^ | 0.17 | 2.06 |
| 5-Methoxyluteolinidin (mg/g) |  | 1.35^a^ | 0.55^a^ | 0.68^a^ | 0.35 | 0.43 |
| 7-Methoxyapigeninidin (mg/g) |  | 0.60^a^ | 1.05^a^ | 0.40^a^ | 0.17 | 0.96 |
| Luteolin (mg/g) |  | 0.26^a^ | 0.44^a^ | 0.24^a^ | 0.03 | 0.16 |
| Apigenin (mg/g) |  | 0.18^a^ | 0.83^a^ | 0.42^a^ | ND | 0.09 |

Different superscript letters in a row indicate significantly different means (p < 0.05).

^*^Colors with a single sorghum genotype; ^†^Equals to total monounsaturated fatty acid content.

ABTS: ABTS**^•^**^+^ scavenging activity; AI: Antioxidant index; DPPH: DPPH^•^ scavenging activity; FRAP: Ferric reducing antioxidant power.
